# Diagnosis of Neonatal Transient Tachypnea and Its Differentiation From Respiratory Distress Syndrome Using Lung Ultrasound

**DOI:** 10.1097/MD.0000000000000197

**Published:** 2014-12-12

**Authors:** Jing Liu, Yan Wang, Wei Fu, Chang-Shuan Yang, Jun-Jin Huang

**Affiliations:** From the Department of Neonatology and NICU of Bayi Children's Hospital (JL, YW, WF, C-SY, J-JH), Beijing Military General Hospital, Beijing; and Graduate School of Southern Medical University (YW, WF), Guangzhou, China.

## Abstract

Transient tachypnea of the newborn (TTN) is one of the most common causes of perinatal dyspnea and is traditionally diagnosed by chest x-ray. This study aimed to explore the diagnostic value of lung ultrasonography (LUS) for TTN as well as differentiate it from respiratory distress syndrome (RDS) by using LUS.

From January 2013 to February 2014, 60 infants who were diagnosed with TTN based on medical history, clinical manifestations, arterial blood gas analysis, and chest radiography were recruited to the study group. During the same period, 40 hospitalized neonates with nonlung diseases and 20 patients with RDS were recruited to the control group. In a quiet state, infants were placed in the supine, lateral, or prone position for the examination. Each lung of every infant was divided into 3 regions: the anterior, lateral, and posterior regions as bordered by the anterior axillary and posterior axillary lines. The probe was placed perpendicular to the ribs. Each region of both the lungs was carefully scanned.

The common ultrasonographic manifestations of TTN were double-lung point (DLP), interstitial syndromes or white lungs, pleural line abnormalities, and A-line disappearance. A small number of infants (20%) with TTN exhibited pleural effusions, whereas the main ultrasonographic manifestation of RDS was lung consolidation with air bronchograms, which does not occur in TTN. The sensitivity and specificity of DLP for the diagnosis of TTN were 76.7% and 100%, respectively.

LUS can accurately and reliably diagnose TTN. The DLP and lung consolidation possess great value in the diagnosis and differential diagnosis of TTN with RDS. Thus, we believe that LUS can be widely used in neonatal intensive care units.

## INTRODUCTION

Transient tachypnea of the newborn (TTN) is also called wet lung, and it is one of the most common causes of perinatal dyspnea. The incidence rates of TTN are 4.0% to 5.7% among term infants and 10.0% among premature infants.^1^ Although TTN rarely causes neonatal death, it is necessary to correctly differentiate TTN from other causes of dyspnea, such as respiratory distress syndrome (RDS), meconium aspiration syndrome (MAS), and pneumonia, to correctly manage infants with TTN and dyspnea. In the past, the diagnosis of TTN was primarily based on medical history, clinical manifestations, findings of arterial blood gas analysis, and chest radiography (CXR) examinations. As a promising technique, lung ultrasonography (LUS) has been successfully utilized in the diagnosis and differential diagnosis of neonatal, childhood, and adult lung diseases.^2–12^ However, as one of the most common causes of neonatal respiratory difficulty, it is hard to distinguish TTN from RDS clinically even by clinical manifestation and by CXR. As discussed in the present study, we have found that LUS is accurate and reliable for the early diagnosis of TTN and is also very useful to easily distinguish TTN from RDS.

## PATIENTS AND METHODS

### Patients

Prior to conducting this study, we received informed consent from the infant's parents and approval from the Ethics Committee of the Beijing Military General Hospital, Beijing, China. From January 2013 to February 2014, 60 infants were hospitalized at the neonatal intensive care unit (NICU) of the Bayi Children's Hospital affiliated with the Beijing Military General Hospital and were diagnosed with TTN based on medical history, clinical manifestations, arterial blood gas analysis, and CXR examination. Among these 60 children, there were 20 premature infants (12 males and 8 females) with gestational ages (GAs) ranging from 27^+3^ to 36^+2^ weeks; 12 of the infants were delivered by cesarean section and 8 were delivered vaginally. The birth weights of these 20 premature infants ranged from 1080 to 3980 g. There were also 40 term infants among the 60 infants (22 males and 18 females), with GAs ranging from 37^+6^ to 41^+6^ weeks; 25 of the infants were delivered by cesarean section and 15 were delivered vaginally. The birth weights of these 40 infants ranged from 2000 to 4150 g. The control group included 2 subgroups. Subgroup 1 included 40 newborns with no lung disease who were hospitalized during the same period of time. In subgroup 1, 24 infants were delivered by cesarean section and 16 were delivered vaginally. Among these 40 newborns, there were 15 premature infants (9 males and 6 females), with GAs ranging from 28 to 36^+1^ weeks. The birth weights of these infants ranged from 1100 to 3550 g; 7 premature infants were delivered by cesarean section and 8 premature infants were delivered vaginally. There were also 25 term infants (15 males and 10 females) in this subgroup, with GAs ranging from 37 to 41^+1^ weeks, and birth weights ranging from 2010 to 3950 g. Subgroup 2 included 20 patients with RDS, among whom there were 13 premature infants and 7 term infants. The GAs ranged from 27^+5^ to 40 weeks, and the birth weights ranged from 980 to 4440 g.

### Methods

#### Equipment

GE Volusion i or Volusion E8 (GE Medical Systems, Milwaukee, USA) ultrasound instruments and a linear array probe with a frequency of 9.0–12.0 MHz were used in this study.

#### Examination Method

In a quiet state, the infants were placed in the supine, lateral, or prone position for the scan. Each lung was divided into 3 regions—anterior, lateral, and posterior regions—by the anterior and posterior axillary line. The probe was perpendicular to the ribs. Each region of both lungs was carefully scanned.

#### Observation Indexes

The observation indexes included pleural lines, A-lines, B-lines, interstitial syndrome, white lung, lung consolidation with air bronchograms or fluid bronchograms, double-lung point (DLP), and pleural effusion, which were defined as follows.^8–14^ Pleural line: the regular echogenic line under the superficial layers of the thorax moving continuously during respiration, while abnormal pleural lines refer to the pleural line disappearance, thickening, irregularity, or a coarse and indistinct appearance; A-line: a series to echogenic, horizontal, parallel lines equidistant from one another below the pleural line; B-lines: also known as ultrasound lung comets, hyperechoic narrow-based artifacts spreading similar to laser rays from the pleural line to the edge of the screen; Interstitial syndrome: the presence of more than 3 B-lines in every examined area; White lung: defined as the presence of compact B-lines in the 6 areas without horizontal reverberation; Lung consolidation: areas of hepatization with the presence of air bronchograms and/or fluid bronchograms; DLP: because of a difference in the severity or nature of the pathological changes in different areas of the lung, a longitudinal scan shows a clear difference between the upper and lower lung fields; this sharp cutoff point between the upper and lower lung fields is known as the DLP; and Pleural effusion: anechoic-dependent collections limited by the diaphragm and the pleura.

#### Statistical Analysis

The Statistical Package for the Social Sciences 17.0 software (SPSS, Inc, Chicago, IL) was used to statistically analyze the data. Fisher exact test was used to compare the positive rates of the ultrasound test results for the newborns in each group, and the specificity and sensitivity of the main test results for the diagnosis of TTN were calculated based on these results. A value of *P* < 0.05 indicates a statistical difference. The information reported has been evaluated by an expert.

## RESULTS

### Normal Neonatal Lung Ultrasonic Manifestations

**FIGURE 1:**
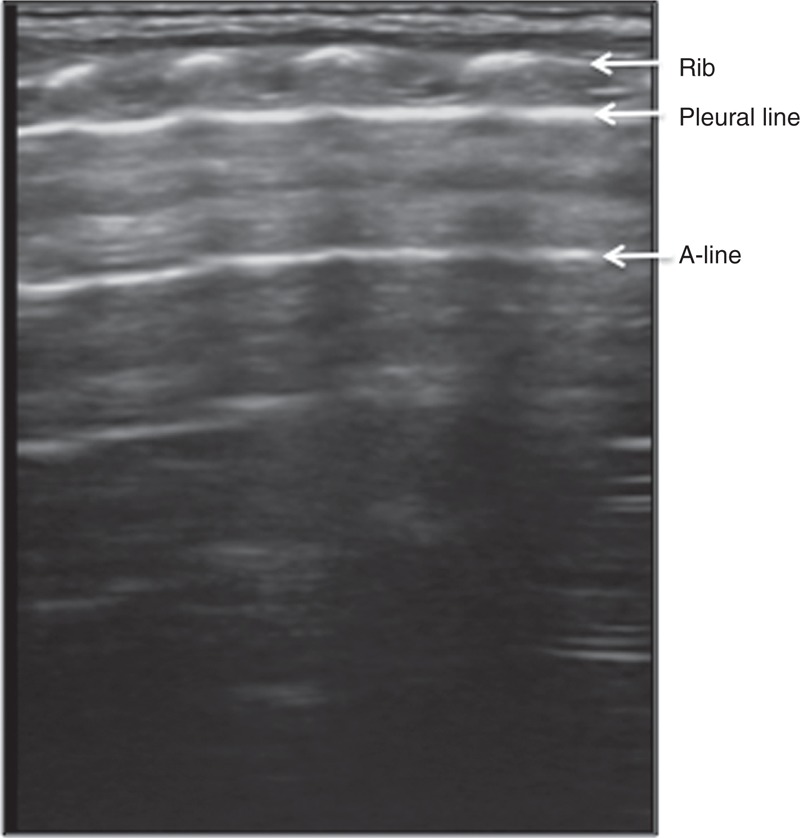
Normal neonatal lung ultrasonic manifestations. The normal neonatal lung ultrasonic manifestations were as follows. The lung tissue was overall hypoechoic, with pleural lines and A-lines that were smoothly, clearly and regularly hyperechoic. There were no manifestations such as interstitial lung syndrome, pulmonary consolidation, double-lung point, or pleural effusion.

The normal lung tissue was hypoechoic. The pleural lines and A-lines were smoothly, clearly, and regularly hyperechoic, and they were equidistantly distributed in parallel. There were no B-lines (72 hours after birth) or only a few (within 72 hours after birth). There were no manifestations such as interstitial lung syndrome, pulmonary consolidation, DLP, and pleural effusion (Figure 1).

### Lung Ultrasonic Manifestations of TTN

**FIGURE 2:**
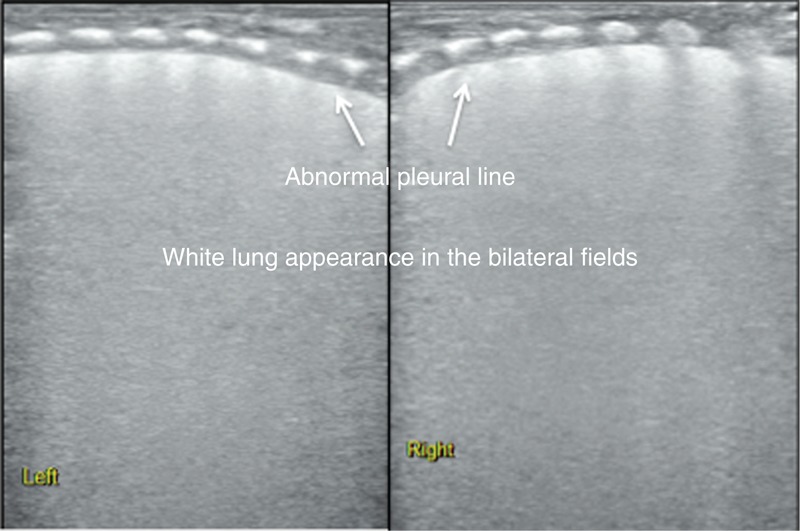
Ultrasonic manifestation of TTN: interstitial lung syndrome and white lung. The infant above had a GA of 27^+3^ wk and was delivered vaginally; the infant had a birth weight of 1020 g. The infant was admitted to the hospital after suffering from dyspnea for 6 h and was diagnosed with TTN based on an arterial blood gas analysis and CXR manifestations. Lung ultrasonography showed thickening and fuzziness of the pleural lines in both the lungs as well as A-line disappearance and white lung appearance. CXR = chest radiography, GA = gestational age, TTN = transient tachypnea of the newborn.

**FIGURE 3:**
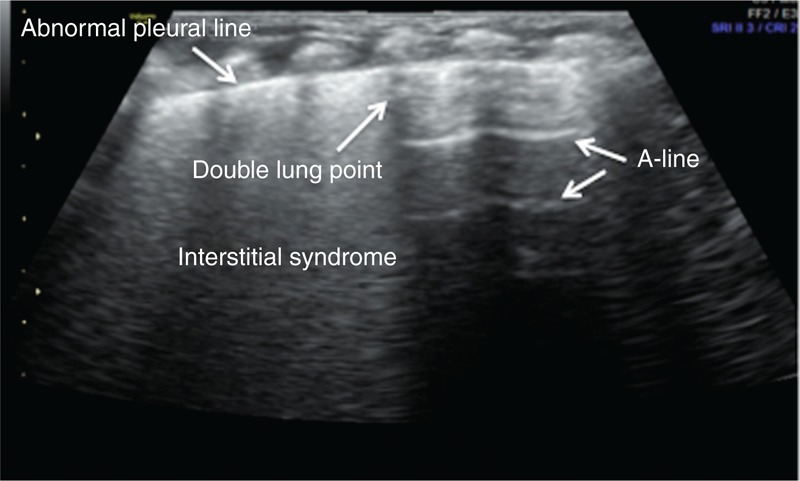
Ultrasonic manifestation of TTN: partial A-line disappearance. The infant above had a GA of 31 wk and was delivered vaginally; the infant had a birth weight of 1550 g. The infant was admitted to the hospital 5 h after birth after suffering from dyspnea for 5 h. Lung ultrasonography showed clear double-lung point, interstitial lung syndrome, and disappearance of the A-line at the interstitial lung syndrome site (there were A-lines at other sites). GA = gestational age, TTN = transient tachypnea of the newborn.

**FIGURE 4:**
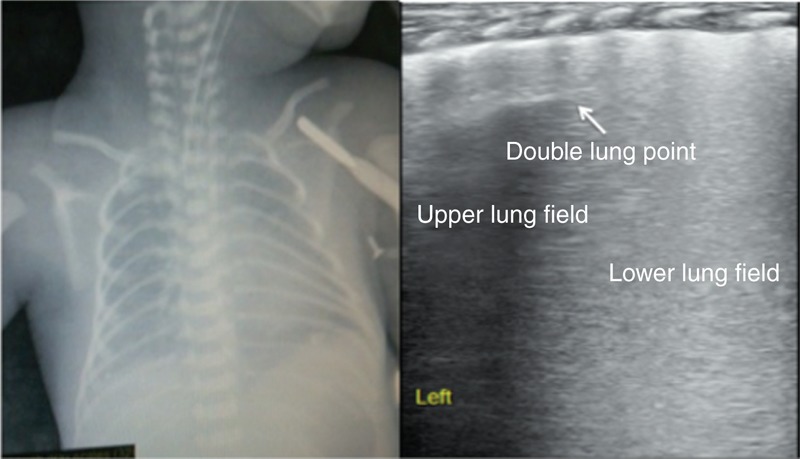
Ultrasonic manifestation of TTN: double-lung point. The infant above had a GA of 28^+1^ wk and was delivered vaginally; the infant had a birth weight of 1150 g. The infant was admitted to the hospital after suffering from dyspnea for 4 h. Lung ultrasonography showed clear double-lung point, interstitial lung syndrome, and fuzziness of the pleural lines. GA = gestational age, TTN = transient tachypnea of the newborn.

**FIGURE 5:**
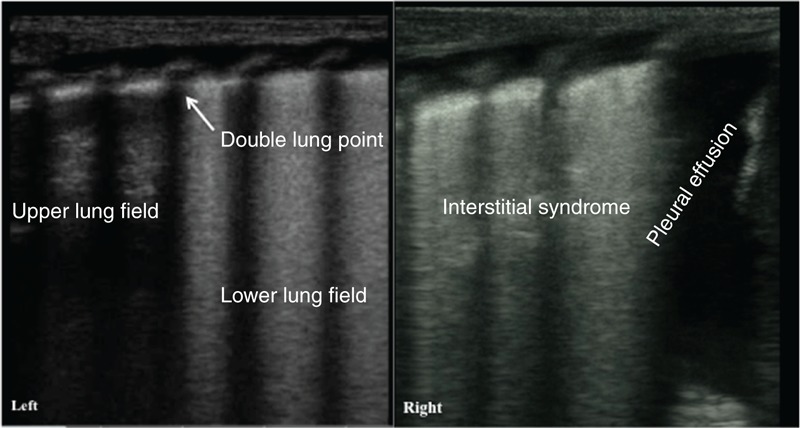
Ultrasonic manifestation of TTN: pleural effusion. The infant above had a GA of 39 wk and was delivered by cesarean section; the infant had a birth weight of 3980 g. The infant was admitted to the hospital after suffering from dyspnea for 5 h. Lung ultrasonography showed the disappearance of the pleural lines in both the lungs, A-lines, and interstitial syndrome in the left lung field and pleural effusion in the right lung. This case also demonstrated that the degree of the injury in each of the lungs in a child with TTN could be different. GA = gestational age, TTN = transient tachypnea of the newborn.

The main lung ultrasonic manifestations of TTN were as follows. Interstitial lung syndrome: it was observed in all of the infants. For severe patients, white lung was observed (Figure 2); Pleural line abnormalities: these were observed in all of the patients with TTN. The abnormalities included thickening, fuzziness, and disappearance (Figure 2); Partial or complete disappearance of A-lines: in infants with severe interstitial lung syndrome, the A-lines completely disappeared; otherwise, the A-lines only partially disappeared (Figures 2 and 3); DLP: it was observed in 46 infants (76.7%) (Figure 4); Pleural effusion: this, either unilateral or bilateral, was observed in 12 infants (20%) (Figure 5); Inconsistent ultrasonic manifestations of bilateral lung fields: not only were the ultrasonic manifestations of bilateral lung fields different, but the ultrasonic features of different lung fields within the same lung could also differ, which was related to the degree of lung lesion (Figure 5); and no lung consolidation was observed in any of the infants (Table 1).

**TABLE 1:**
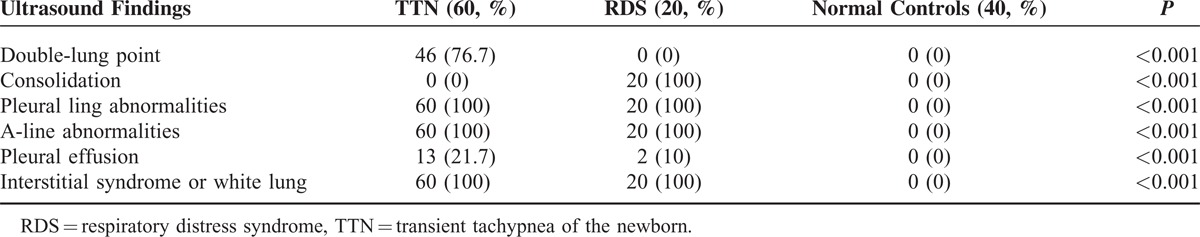
Ultrasound Findings in TTN, RDS, and Normal Controls (n, %)

### Sensitivity and Specificity of DLP for the Diagnosis of TTN

**FIGURE 6:**
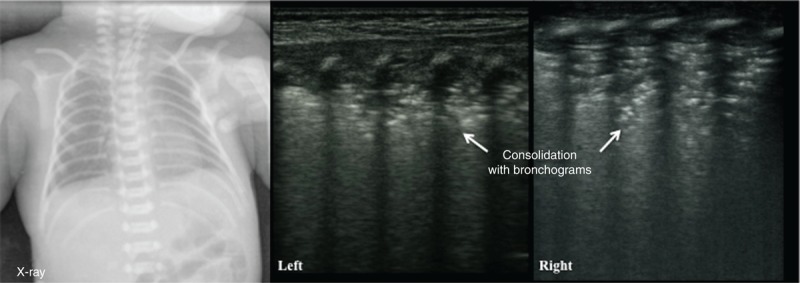
Ultrasonic manifestation of RDS: pulmonary consolidation with air bronchograms. The infant above had a GA of 39^+1^ wk and was delivered by cesarean section; the infant had a birth weight of 4000 g. The infant was admitted to the hospital because of dyspnea for 4 h. The CXR indicated RDS. LUS showed large-area lung consolidation with significant air bronchograms in bilateral lungs and the disappearance of the pleural lines and A-lines. CXR = chest radiography, GA = gestational age, LUS = lung ultrasonography, RDS = respiratory distress syndrome.

**FIGURE 7:**
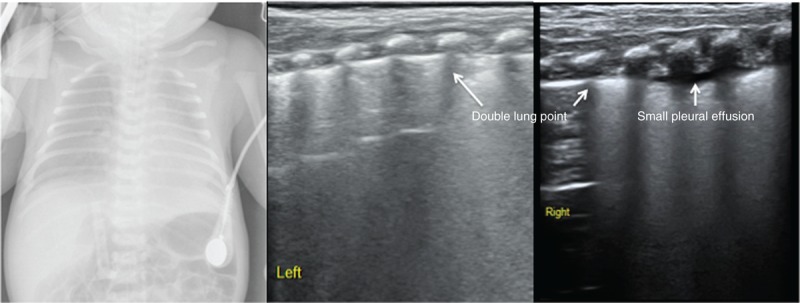
Differentiation between TTN and RDS. The infant above had a GA of 38 wk and was delivered by cesarean section; the infant had a birth weight of 2750 g. The infant started to experience progressive dyspnea soon after birth. The infant was diagnosed with RDS based on medical history and clinical and CXR manifestations. However, lung ultrasonography only showed pleural line abnormalities, white lung, pleural effusion, and A-line disappearance without lung consolidation. Therefore, the infant suffered from TTN rather than RDS. The clinical course of the infant was also consistent with TTN. CXR = chest radiography, GA = gestational age, RDS = respiratory distress syndrome, TTN = transient tachypnea of the newborn.

According to the literature and the results of this study, the most important ultrasonic manifestation of RDS was lung consolidation with air bronchograms (100%); in addition, pleural line abnormalities, the disappearance of A-lines, and interstitial syndrome could also be manifested, but DLP was not found in patients with RDS (Figure 6). DLP was only observed in infants with TTN and not in infants with RDS^2,4,7,12,14^; therefore, the sensitivity and specificity of DLP for the diagnosis of TTN were calculated in this study. The results showed that the sensitivity of DLP for the diagnosis of TTN was 76.7%, but the specificity was 100% (Table 2). Because DLP is a specific feature of TTN and lung consolidation is observed only in patients with RDS, DLP and lung consolidation with air bronchograms are the most important features for differentiating TTN from RDS using LUS (Figure 7).

**TABLE 2:**
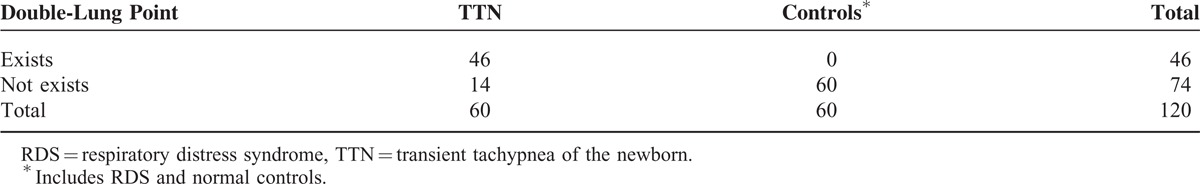
Sensitivity and Specificity of Double-Lung Point to Diagnoses of TTN

## DISCUSSION

Dyspnea is a common severe acute disease among newborns that must be treated timely and accurately. Many common lung diseases can cause dyspnea in newborns, including TTN, RDS, MAS, pneumonia, pneumothorax, pulmonary hemorrhage, and congenital diaphragmatic hernia, among others. Of these diseases, TTN is the most common cause of neonatal dyspnea. Approximately, 1/3 to 1/2 of dyspnea in newborns is caused by TTN.^15^ The timely and accurate diagnosis of this diseases is crucial for correct treatment and to improve prognosis. The results of this study further demonstrated that the use of LUS to diagnose TTN was simple, accurate, and reliable for an early diagnosis.^12^ According to the results of this study, the main ultrasonic imaging features of TTN include DLP, interstitial lung syndrome/white lung, pleural line abnormalities, and A-line disappearance. Certain infants (20%) suffered from pleural effusion to different degrees. The sensitivity and specificity of DLP for the diagnosis of TTN were 76.7% and 100%, respectively, in contrast to previous research results that reported that the sensitivity of DLP for the diagnosis of TTN was 100%.^12^ This disagreement might be due to different disease severity. According to our experience, white lung appearance is often observed in relatively severe patients with TTN, whereas DLP is more often observed in infants with less-severe TTN.

The main pathological mechanism of TTN is increased water content in the lung tissues, which is manifested as interstitial syndrome on ultrasonography. Therefore, interstitial syndrome is the most important and common ultrasonic feature of TTN; in patients with severe disease, TTN is manifested as white lung or even pleural effusion, which was observed in 20% of the infants in this study. However, interstitial syndrome can also be observed in RDS, pneumonia, and pulmonary edema occurring because of other causes^2,4–7,13^; therefore, interstitial syndrome is not a specific feature of TTN. Similarly, pleural line and A-line abnormalities are not specific features of TTN.

It was also discovered in this study that the ultrasonic features of different lung fields could be different in patients with TTN. DLP was manifested on one side and interstitial lung syndrome or white lung on the other side; pleural effusion could exist on one side of the thorax alone. This phenomenon indicated that the water content of the lung tissue (ie, the degree of pulmonary edema) in different regions was inconsistent in patients with TTN. Therefore, LUS is beneficial for further understanding lung diseases such as TTN.

It is estimated that 77% of infants with TTN are clinically misdiagnosed with RDS.^1^ In particular, patients with dyspnea whose CXR examinations reveal a white lung appearance are often diagnosed with RDS. LUS is beneficial for differentiating TTN from RDS. Based on the results of this study in combination with literature reports,^2,4,7,16^ the primary ultrasonic feature of RDS is lung consolidation with air bronchograms without DLP, while the most specific ultrasonic feature of TTN is DLP without lung consolidation.^12^ Therefore, LUS is not only useful to diagnose TTN and RDS but also valuable to differentiate TTN from RDS (Figure 7).

## CONCLUSIONS

In summary, the results of this study further demonstrate that LUS can accurately and reliably diagnose TTN. In addition, LUS is valuable for distinguishing among causes of neonatal dyspnea, especially for the differentiation of TTN and RDS. Last, LUS can also be used for differentiating the pulmonary causes of long-term oxygen dependence in patients with bronchopulmonary dysplasia,^17^ and it has advantages that cannot be duplicated by chest computed tomography or CXR examination.^18^ Therefore, LUS could be widely used in NICUs as the first-choice diagnostic measure for lung diseases.^19,20^ Certainly, LUS techniques require properly trained staff with the appropriate equipment before it can routinely be used clinically.
